# Loss of *ANK3* Function Causes a Recessive Neurodevelopmental Disorder with Cerebellar Ataxia

**DOI:** 10.1002/mds.30324

**Published:** 2025-08-04

**Authors:** Reza Maroofian, Giulia Spoto, Dalila Moualek, Maha S. Zaki, Asthik Biswas, Felice D'Arco, Sajjad Biglari, Pooneh Nikuei, Joseph G. Gleeson, Meriem Tazir, Lamia Ali Pacha, Henry Houlden

**Affiliations:** ^1^ Department of Neuromuscular Diseases University College London, Queen Square, Institute of Neurology London UK; ^2^ Department of Biomedical Sciences Dental Sciences & Morpho‐Functional Imaging, University of Messina Messina Italy; ^3^ Service de Neurologie, CHU Mustapha Bacha Alger Algeria; ^4^ Université Benyoucef Benkhedda Alger Algeria; ^5^ Clinical Genetics Department Human Genetics and Genome Research Institute, National Research Centre Cairo Egypt; ^6^ Medical Genetics Department Armed Forces College of Medicine Cairo Egypt; ^7^ Pediatric Neuroradiology Unit, Radiology Department Great Ormond Street Hospital for Children London UK; ^8^ Department of Genetics and Molecular Biology Isfahan University of Medical Sciences Isfahan Iran; ^9^ Molecular Medicine Research Center, Hormozgan Health Institute Hormozgan University of Medical Sciences Bandar Abbas Iran; ^10^ Rady Children's Institute for Genomic Medicine San Diego California USA; ^11^ Department of Neurosciences and Pediatrics University of California, San Diego San Diego California USA

**Keywords:** *ANK3*, cerebellar ataxia, cerebellar atrophy, neurodevelopmental disorder

## Abstract

**Background:**

*ANK3* encodes ankyrin‐G, a key scaffolding protein essential for neuronal function. While both monoallelic and biallelic *ANK3* variants have been linked to neurodevelopmental disorders (NDDs), existing evidence for their pathogenicity and clinical correlation remains limited and heterogeneous.

**Objective:**

To delineate the clinical features associated with biallelic *ANK3* predicted loss‐of‐function (pLOF) variants.

**Methods:**

We employed exome sequencing, Sanger validation, detailed clinical phenotyping, and extensive international data sharing to identify patients with biallelic *ANK3* variants.

**Results:**

We describe five individuals from three unrelated consanguineous families with segregating homozygous *ANK3* pLOF variants. These patients presented with a relatively consistent phenotype comprising developmental delay, intellectual disability, hypotonia, variable epilepsy, and cerebellar signs including ataxia, tremor, and dysarthria. Among the three patients for whom brain magnetic resonance imaging was available, cerebellar atrophy was observed, predominantly affecting the superior vermis and cerebellar hemispheres. These clinical findings align with murine models lacking the cerebellar ankyrin‐G isoform, which similarly exhibit ataxic features and high cerebellar *ANK3* expression.

**Conclusion:**

Our findings support a recognizable NDD with non‐progressive cerebellar ataxia linked to biallelic *ANK3* pLOF variants. © 2025 The Author(s). *Movement Disorders* published by Wiley Periodicals LLC on behalf of International Parkinson and Movement Disorder Society.

Ankyrins—Ankyrin‐R, ‐B, and ‐G—are a family of large scaffolding proteins encoded by the *ANK1*, *ANK2*, and *ANK3* genes, respectively. They act as critical adaptors by anchoring integral membrane proteins to the spectrin–actin cytoskeleton. Although *ANK1* is predominantly expressed in erythrocytes, where pathogenic variants are associated with hereditary spherocytosis type 1 (OMIM #182900), it was recently found also in the brain, where it scaffolds voltage‐gated potassium channels.^1^, ^2^ In contrast, *ANK2* and *ANK3* are more widely expressed, including in the central nervous system: ankyrin‐B (*ANK2*) plays a key role in ion channel organization, with pathogenic variants linked to cardiac arrhythmia (OMIM #600919) and several neurodevelopmental disorders (NDDs); ankyrin‐G (*ANK3*) serves as a master organizer of the axon initial segment and the nodes of Ranvier, facilitating action potential initiation and propagation along the axon.^2^, ^3^, ^4^

Biallelic predicted loss‐of‐function (pLoF) variants in *ANK3* have been associated with autosomal recessive intellectual developmental disorder 37 (OMIM #615493), based on only two previously reported families with very limited clinical characterization. Additionally, several biallelic missense variants across this large protein, 4377 amino acids, have been reported, though the majority are classified as variants of uncertain significance (VUS) or likely benign/benign, and frequently associated with non‐specific and heterogenous neurodevelopmental phenotypes. As such, the gene–disease validity and phenotypic spectrum associated with recessive *ANK3* variants remain poorly defined and under debate.

Monoallelic *ANK3* variants have also been linked to NDDs characterized by developmental delay (DD)/intellectual disability (ID), and psychiatric or behavioral features.^5^, ^6^, ^7^, ^8^, ^9^, ^10^, ^11^, ^12^, ^13^

In this study, we report five individuals from three unrelated families carrying biallelic pLoF *ANK3* variants, presenting with a distinct phenotype prominently featuring cerebellar signs. To better delineate the clinical spectrum of recessive *ANK3*‐related disease, we also reviewed previously reported cases and propose a refined phenotype associated with biallelic *ANK3* disruption.

## 1 Methods

### 1.1 Patient Recruitment and Clinical and Genetic Investigation

Affected individuals with biallelic *ANK3* variants were identified through international collaborations. Exome sequencing was performed on genomic DNA extracted from blood. The candidate variants were confirmed after filtering and interpretation according to the American College of Medical Genetics and Genomics and the Association for Molecular Pathology (ACMG‐AMP) guidelines,^14^ and segregation analysis was performed by Sanger sequencing. Brain magnetic resonance imaging (MRI) studies were reviewed in consensus by two experienced pediatric neuroradiologists.

## 2 Results

### 2.1 Clinical Characterization of Five Patients from Three Families with *ANK3* Deficiency

Affected individuals from Family 1 are two Algerian sisters born to consanguineous healthy parents. Patient 1 was born after a full‐term pregnancy, with a birthweight of 2.8 kg. She displayed nystagmus and alternating strabismus from the age of 3 months, a DD without regression, and a balance disorder; she could walk only with support. From the age of 3 years she developed myoclonic and generalized tonic clonic seizures treated with valproate. Dysmorphic features included high forehead, telecanthus, and pointed chin. Neurological examination performed at 10 years revealed a non‐progressive, severe, static and kinetic cerebellar syndrome with action tremor from the age of 3 years, generalized hypotonia associated with kyphosis, scoliosis, and flat valgus feet. She presented vivid osteotendinous reflexes and bilateral Babinski sign. Language was dysarthric, while clinical assessment suggested a mild ID.

**FIG. 1:**
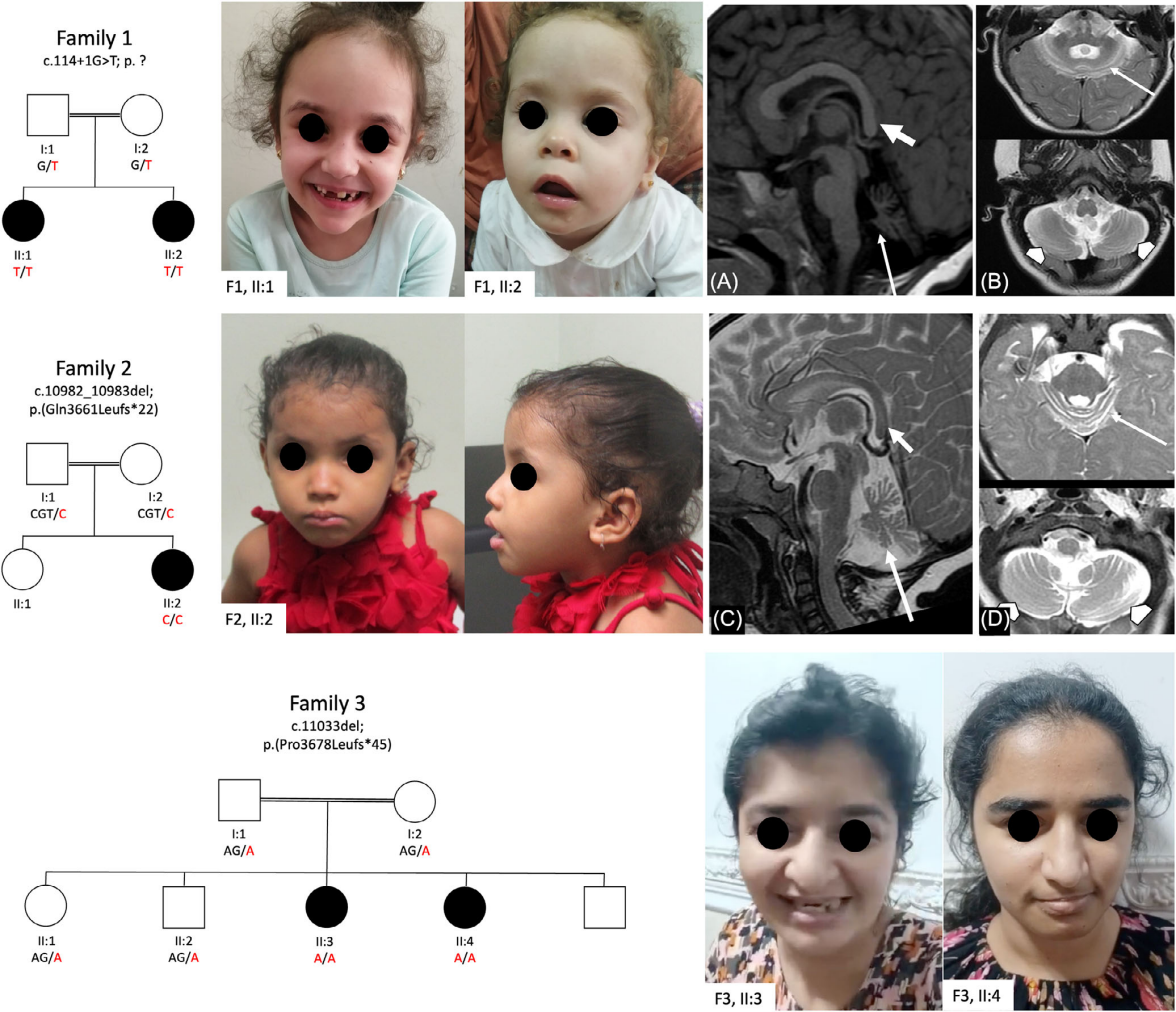
Pedigrees of Families 1, 2, and 3; facial photos of Patients 1–5 showing shared dysmorphic features. Magnetic resonance imaging scans of Patients 1 and 2 showing dysmorphic corpus callosum (short arrows in A and C) and cerebellar atrophy with superior predominance (long arrows in A, B,C, and D) and relatively spared inferior hemispheres (arrowheads in B and D). [Color figure can be viewed at wileyonlinelibrary.com]

Brain MRI showed cerebellar atrophy involving the vermis and the cerebellar hemispheres with widening of the fissures and “bright cortex” on T2‐weighted images; the atrophy was more prominent in the superior lobules of the vermis and hemispheres with relative sparing of the inferior aspects (Fig. 1A,B). Furthermore, the posterior aspect of the corpus callosum was slender and dysmorphic with characteristic “drooping” appearance. Electroencephalography (EEG) displayed generalized spike–wave and polyspike complexes on a theta background activity, worsening during activation procedures. Electromyoneurography, abdominal ultrasound, heart examination, and fundus oculi were normal.

Patient 2 was born after a full‐term, uneventful pregnancy. She presented with neonatal hypotonia and displayed moderate DD without regression. On physical examination she exhibited joints hyperlaxity, kyphosis, flat valgus feet, and dysmorphic features (high forehead, telecanthus, epicanthus, depressed nasal bridge, pointed chin, and low‐set ears). Neurological examination performed at 6 years of age found strabismus and cerebellar signs, such as slight truncal ataxia and an action tremor. She could only walk with support. She showed vivid osteotendinous reflexes and spontaneous bilateral Babinski sign. She developed myoclonic seizures from the age of 5 years.

Brain MRI revealed findings remarkably similar to those described in Patient 1 (Fig. 1C,D). EEG displayed epileptic abnormalities. Electromyoneurography, abdominal ultrasound, heart examination, and fundus oculi were normal.

Exome sequencing in both patients revealed a homozygous *ANK3*(NM_020987.5): c.114 + 1G>T variant inherited from the patients’ parents, which involves a splicing site, and is predicted to result in LOF and absence of protein production. The variant is classified as likely pathogenic according to the ACMG‐AMP criteria and was not found in the Genome Aggregation Database (gnomAD).

Patient 3 (Family 2) was briefly mentioned in the supplementary material of a large cohort of recessive pediatric neurogenetic diseases.^15^ She is a 5‐year‐old Egyptian girl, second child born to consanguineous healthy parents after a full‐term, uneventful pregnancy. She displayed infantile hypotonia and DD without regression; she acquired the sitting position at 15 months, walked after 4 years, spoke at 3 years, and attained only five words. On physical examination she exhibited low‐to‐normal growth parameters and dysmorphic features (mask‐like face, high forehead, broad nasal root, epicanthus, prominent pillars, V‐shaped upper lip, mild retruded mandible, and low‐set ears). Neurological examination found generalized hypotonia, abnormal upward gaze, and prominent cerebellar signs, such as postural instability, ataxic gait, dysarthria, and static and kinetic tremor. She displayed normal osteotendinous reflexes and mute plantar response. The patient also displayed hyperactive behavior, bruxism, and sleep disturbances.

Brain MRI revealed, similar to the aforementioned cases, cerebellar atrophy with superior vermis and hemispheres being more involved. The corpus callosum was thin posteriorly but there was no “drooping” appearance. Mild posterior periventricular white matter hyperintensities were also noted. EEG, electromyoneurography, heart examination, and metabolic screening were normal.

Exome sequencing revealed a homozygous *ANK3* (NM_020987.5): c.10981_10982del variant, inherited from both parents, which causes a frameshift mutation, and is predicted to result in LOF and production of a truncated protein (p.Gln3661Valfs*22). The variant is classified as likely pathogenic according to the ACMG‐AMP criteria and was not found in gnomAD.

Family 3, including two affected Iranian sisters, was briefly reported in the supplementary material of a large cohort of families with intellectual disability.^6^ Patient 4 is a 27‐year‐old woman born to consanguineous healthy parents after a full‐term pregnancy. She presented with infantile hypotonia and DD without regression; she acquired the sitting position at 4 years, walked at 5 years, and never attained speech. She had severe ID, and neurological examination found non‐progressive ataxic gait, postural instability, and bilateral Babinski sign. She also displayed dysmorphic features (telechantus, downward‐slanting palpebral fissures, protruded mandible, and low‐set ears). She had epilepsy onset at 3 days of age and displayed sleep disturbances. Brain MRI, EEG, or electromyoneurography were not available.

Patient 5 is a 23‐year‐old woman born after a full‐term pregnancy. She showed infantile hypotonia and DD without regression; she acquired the sitting position at 14 months, walked at 42 months, and never attained speech. She had severe ID, but neurological examination was normal except for bilateral Babinski sign. She also displayed mild dysmorphisms (downward‐slanting palpebral fissures and low‐set ears). Brain MRI, EEG, or electromyoneurography were not available.

Exome sequencing revealed a homozygous *ANK3* (NM_020987.5): c.11033del variant, inherited from both parents, which causes a frameshift mutation, and is predicted to result in LOF and production of a truncated protein (p.Pro3678Leufs*45). The variant is classified as likely pathogenic according to the ACMG‐AMP criteria and was not found in gnomAD.

## 3 Discussion

*ANK3* encodes a large protein of 4377 amino acids, making the detection of variants common during genetic testing; however, not all of these variants are clinically relevant. To date, 16 biallelic (summarized in Tables 1 and S1 for pLOF and missense variants, respectively) and 23 monoallelic (Table S2) *ANK3* variants have been reported in the literature, associated with heterogeneous phenotypes.^5^, ^7^, ^8^, ^9^, ^10^, ^11^, ^16^, ^17^, ^18^, ^19^, ^20^, ^21^, ^22^ Nevertheless, many have been classified under ACMG‐AMP criteria as VUS, likely benign, or benign, highlighting the challenges in variant interpretation for this gene.

**TABLE 1:** Clinical, genetic, and neuroimaging features of patients carrying biallelic loss‐of‐function variants of *ANK3*

Reference	This report Patient 1	This report Patient 2	This report Patient 3	This report Patient 4	This report Patient 5	Iqbal et al.^5^	Iqbal et al.^5^	Iqbal et al.^5^
Sex, age on examination	F, 10 y	F, 6 y	F, 5 y	F, 27 y	F, 23 y	F, 25 y	F, 22 y	M, 18 y
Ethnicity	Algerian	Algerian	Egyptian	Iranian	Iranian	Pakistani	Pakistani	Pakistani
Genetic variant	c.114 + 1G>T p.?	c.114 + 1G>T p.?	c.10981_10982del p.Q3661Vfs*22	c.11033del p.P3678Lfs*45	c.11033del p.P3678Lfs*45	c.10995del p.T3666Lfs*2	c.10995del p.T3666Lfs*2	c.10995del p.T3666Lfs*2
Movement disorders	Ataxia, dysmetria, static and kinetic tremor, nystagmus	Ataxia and action tremor	Ataxia, mild static and kinetic tremor, abnormal upward gaze	Ataxia	No	NR	NR	NR
Muscular tone	Hypotonia	Hypotonia	Hypotonia	Infantile hypotonia	Infantile hypotonia	Hypotonia + spasticity	Hypotonia + spasticity	Hypotonia + spasticity
ID severity	Mild	Moderate	Severe	Severe	Severe	Moderate	Moderate	Moderate
Language disorder	Dysarthria	Speech delay	Dysarthria	Absent speech	Absent speech	Speech delay	Speech delay	Speech delay
Behavioral disturbances	No	Hyperactivity	Hyperactivity, bruxism	No	No	Hyperactivity, unspecified behavioral problems	Hyperactivity, unspecified behavioral problems	Hyperactivity, unspecified behavioral problems
Sleep disorder	No	No	Disturbed sleep, bruxism	Sleep disturbances	No	Diurnal somnolence	Diurnal somnolence	Diurnal somnolence
Epilepsy	GTCS and myoclonic seizures	Myoclonic seizures	No	Yes (unspecified seizures)	No	No	No	GTCS
Brain neuroimaging	Cerebellar atrophy involving predominantly the superior aspects of the vermis and hemispheres, dysmorphic CC with slender and “drooping” posterior body and splenium	Cerebellar atrophy involving predominantly the superior aspects of the vermis and hemispheres, dysmorphic CC with slender and “drooping” posterior body and splenium	Cerebellar atrophy involving predominantly the superior aspects of the vermis and hemispheres, thin posterior CC, mild posterior PWM hyperintensities	NR	NR	NR	Normal CT	Normal CT
Dysmorphic features	Yes	Yes	Yes	Yes	Yes	No	No	No

Abbreviations: CC, corpus callosum; CT, computed tomography; F, female; GTCS, generalized tonic clonic seizures; ID, intellectual disability; M, male; NR, not reported; PWM, periventricular white matter; y, years.

Establishing a precise genotype–phenotype correlation requires a comprehensive and detailed clinical characterization to ensure that only definitively pathogenic variants are considered. Applying this approach, we describe five patients with a consistent and distinctive phenotype and reviewed previously reported biallelic pLOF variants to further characterize *ANK3*‐related NDD.

Across patients with biallelic pLOF variants, a consistent phenotype emerged, characterized by hypotonia and DD followed by ID of varying severity (mild in 1/8, moderate in 4/8, severe in 3/8), as well as language disorders (speech delay in 6/8, dysarthria in 2/8). Among the present cohort, most patients (4/5) demonstrated cerebellar symptoms including ataxia (4/5), tremor (3/5), and cerebellar atrophy (3/5), which predominantly involved the superior vermis and cerebellar hemispheres. Additional findings included a slender posterior body and splenium of the corpus callosum.

While cerebellar atrophy is a relatively non‐specific MRI finding, it can suggests an underlying neurogenetic disorder in the appropriate clinical context.^23^ The presence of “bright cortex” in an atrophic cerebellum (i.e., T2‐weighted hyperintensity) is common to several neurogenetic disorders and can aid in differentiating these from conditions with cerebellar atrophy but without hyperintensity.^24^ Radiologically, the combination of a cranio‐caudal gradient of cerebellar atrophy, a small posterior corpus callosum, and bright cerebellar cortex may serve as a diagnostic clue in the appropriate clinical context.

It is also worth noting that in the patients from the Pakistani family reported by Iqbal and colleagues^5^ comprehensive neurological assessments were lacking. Although spasticity was mentioned, this does not exclude the possibility of additional cerebellar motor features in those patients.

The clinical picture described here—prominent cerebellar involvement within a broader NDD—overlaps with other early‐onset autosomal recessive conditions (Table S3), including *CA8*, *WDR81*, *ATP8A2*, *GPAA1*, and *VLDLR*‐related disorders.^25^, ^26^, ^27^, ^28^, ^29^, ^30^, ^31^, ^32^, ^33^ Such disorders should be considered in the differential diagnosis of spinocerebellar ataxias and spastic–ataxia syndromes.

This distinct phenotype points to a developmental cerebellar disorder, combining cerebellar motor features (ataxia, dysmetria, tremor, nystagmus, dysarthria) with cognitive and emotional difficulties (DD/ID, hyperactivity, aggressive behavior, and expressive language disorders).^34^ Disruption of cerebellar circuits and reduced vermis volume have been linked to DD, cognitive and language deficits, gross and fine motor impairments, behavioral difficulties, and autistic features.^35^ This broad cerebellar involvement may explain the apparent clinical variability, which nonetheless converges on a recognizable and consistent syndrome.

These findings are consistent with the widespread expression of *ANK3*, which is highly abundant in the cerebellum.^36^ Furthermore, in children with developmental cerebellar disorders, motor delay and hypotonia—as seen in all patients with biallelic pLOF variants—can precede the appearance of ataxic movements.^34^

Progressive ataxia has previously been described by Zhou and colleagues in a mouse model lacking the cerebellar‐specific ankyrin‐G isoform. *ANK3* exon 1b‐null mice developed distinctive cerebellar defects, including abnormal gait, tremor, and reduced locomotion.^37^ In contrast, heterozygous knockout of the giant ankyrin‐G isoforms did not result in an ataxic phenotype, suggesting that hemizygosity may cause milder neuromotor involvement.^38^

A similar clinical pattern has been described in some patients carrying biallelic missense *ANK3* variants, with one patient presenting with ataxia and cerebellar hypoplasia^7^ and another with isolated ataxia.^11^ However, these findings should be interpreted cautiously, as these missense variants are classified as VUS or likely benign under ACMG‐AMP criteria.

Conversely, patients with monoallelic variants consistently showed autistic features in 78% (18/23) of cases, which were not reported among patients with biallelic pLOF variants. These data emphasize the phenotypic differences between monoallelic and biallelic *ANK3* variants and underscore the importance of thorough clinical evaluation to fully delineate the disorder spectrum.

In conclusion, our findings define a novel autosomal recessive neurodevelopmental syndrome with non‐progressive cerebellar ataxia, refining the phenotypic spectrum of ultra‐rare recessive *ANK3*‐related disorders. This study also provides new insights into cerebellar‐specific functions of ankyrin‐G and expands the list of genes implicated in rare autosomal recessive spinocerebellar ataxias.

## Author Roles

(1) Research Project: A. Conception, B. Organization, C. Execution, D. Data Analysis and Interpretation; (2) Manuscript Preparation: A. Writing of the First Draft, B. Review and Critique.

R.M.: 1A, 1B, 1C, 1D, 2B.

G.S.: 1C, 1D, 2A, 2B.

D.M.: 1C, 2B.

M.S.Z.: 1C, 2B.

A.B.: 1D, 2B.

F.D.: 1D, 2B.

S.B.: 1C, 2B.

P.N.: 1C, 2B.

J.G.G.: 1C, 1D, 2B.

M.T.: 1C, 2B.

L.A.P.: 1C, 2B.

H.H.: 1C, 2B.

## Supporting information


**Table S1.** Table Information.

**Table S2.** Table Information.

**Table S3.** Table Information.

## Data Availability

The data that supports the findings of this study are available in the supplementary material of this article.
